# Antitumor activity and safety of camrelizumab combined with apatinib in patients with relapsed or refractory peripheral T-cell lymphoma: An open-label, multicenter, phase II study

**DOI:** 10.3389/fimmu.2023.1128172

**Published:** 2023-04-04

**Authors:** Yanfei Liu, Yuqin Song, Shubo Zuo, Xian Zhang, Hui Liu, Jingwen Wang, Jingbo Wang, Yongjing Tang, Wen Zheng, Zhitao Ying, Lingyan Ping, Chen Zhang, Meng Wu, Jun Zhu, Yan Xie

**Affiliations:** ^1^ Key laboratory of Carcinogenesis and Translational Research (Ministry of Education), Department of Lymphoma, Peking University Cancer Hospital & Institute, Beijing, China; ^2^ Department of Lymphoma, Jilin Guowen Hospital, Siping, China; ^3^ Department of Hematology, Hebei Yanda Lu Daopei Hospital, Langfang, China; ^4^ Department of Hematology, Beijing Hospital, Beijing, China; ^5^ Department of Hematology, Beijing Tongren Hospital, Beijing, China; ^6^ Department of Hematology, Aerospace Central Hospital, Beijing, China

**Keywords:** peripheral T-cell lymphoma, PD-1 inhibitor, apatinib, immunotherapy, camrelizumab

## Abstract

**Introduction:**

The treatment for relapsed/refractory peripheral T-cell lymphoma (r/r PTCL) is suboptimal. This open-label, multicenter, single-arm study aimed to investigate the antitumor activity and safety of camrelizumab (a PD-1 blockade) plus apatinib (an antiangiogenic agent) for patients with r/r PTCL.

**Methods:**

Eligible patients with r/r PTCL were enrolled and received camrelizumab 200 mg intravenously every 2 weeks and apatinib 500 or 250 mg orally once daily, 4 weeks as a cycle. The primary endpoint was overall response rate (ORR).

**Results:**

A total of 20 patients were enrolled and received study medications in the study, with a median number of prior treatment line of 3 (range 1-6). At the cutoff date of March 4, 2022, the median follow-up was 27.2 months (range: 0.5-39.9), and three patients remained on treatment. Six patients had early discontinuation without tumor response evaluation. For all patients, the ORR was 30% (6/20) (95% confidence interval [CI], 11.9% to 54.3%), with two patients (10%) achieving complete response. The median progression-free survival (PFS) and median overall survival for all patients were 5.6 months (95% CI, 1.8 to not reached) and 16.7 months (95% CI, 2.8 to not reached), respectively. Patients with PD-L1 expression ≥50% (3 patients) had a numerically higher ORR and longer median PFS than those with PD-L1 expression < 50% (5 patients). The most commonly reported grade 3 or higher adverse events were hyperlipidemia (15%), hypokalemia (15%) and anemia (15%). No treatment-related deaths occurred.

**Discussion:**

In this study, PD-1 inhibitors plus low-dose antiangiogenic drugs presented preliminary antitumor activity and manageable toxicity in patients with r/r PTCL.

## Introduction

Peripheral T-cell lymphoma (PTCL) is a highly heterogeneous disease that is classified into 27 distinct subtypes according to the 2016 World Health Organization classification of lymphoid neoplasms (1). PTCL accounts for 5% to 10% of all non-Hodgkin lymphoma (NHL) cases in Western countries and approximately 25% in China (2, 3). The relatively prevalent subtypes are peripheral T-cell lymphoma, not otherwise specified (PTCL-NOS), angioimmunoblastic T-cell lymphoma (AITL), anaplastic large cell lymphoma (ALCL) and extranodal NK/T-cell lymphoma, nasal type (ENKTL).

Compared with B-cell lymphoma, most subtypes of PTCL have a worse prognosis, except for ALK-positive ALCL. Currently, the conventional first-line therapy for PTCL is CHOP (cyclophosphamide, doxorubicin, vincristine, and prednisone) or CHOP-like regimens. The ECHELON-2 trial has demonstrated that the addition of brentuximab vedotin, a CD30-directed antibody-drug conjugate, to the first-line therapy significantly improves survival in patients with CD30-positive PTCL (4). However, many patients develop disease progression, and effective treatment is limited for relapsed or refractory (r/r) patients. Except for brentuximab vedotin, a CD30-directed antibody-drug conjugate that has shown promising results against CD30-positive ALCL (5), the effect of other approved drugs, such as pralatrexate and romidepsin, is not satisfactory, with response rates and durations of clinical benefit of 25%-29% and 10-17 months, respectively. Typically, progression-free survival (PFS) was less than four months (6–9), indicating an unmet need for novel therapies.

Over the past few years, immune checkpoint inhibitors (ICIs) have made great advances in oncology. The use of anti-programmed cell death protein-1 (PD-1) has shown remarkable efficacy in tumors, especially in r/r classic Hodgkin lymphoma, with an objective response rate (ORR) of approximately 70% (10). However, the efficacy of anti-PD-1 antibody monotherapy is modest in r/r PTCL, with an ORR of 33-40%, and rapid disease progression poses a concern (11, 12). Thus, it is necessary to further investigate the anti-PD-1 antibodies in r/r PTCL and explore combination therapy to overcome drug resistance.

Apatinib is a novel small-molecule vascular endothelial growth factor receptor-2 (VEGFR-2) tyrosine kinase inhibitor (TKI) that blocks downstream signal transduction by highly selective competition for ATP-binding sites, resulting in strongly inhibited angiogenesis in tumor tissue (13). An exploratory study showed that apatinib monotherapy was tolerable and active, with an ORR of 48%, in r/r NHL patients (14). Anti-angiogenic therapy may enhance immunotherapy by addressing the role of VEGF in immunosuppression. A preclinical study demonstrated that combining a PD-1 inhibitor with apatinib could modulate the tumor microenvironment (TME) and enhance antitumor effects (15). Anti-angiogenic agents can reprogram the TME by normalizing tumor vasculature, promoting antigen presentation, and increasing T cell recruitment and infiltration. Camrelizumab, a potent PD-1 inhibitor, has shown promising activity and tolerable toxicity in different solid tumors and lymphomas (16). Several studies revealed that camrelizumab combined with apatinib was tolerable and demonstrated antitumor activity in treating patients with solid tumors, including cervical cancer, hepatocellular carcinoma, and non-small cell lung cancer (17–19). On the basis of these results, we conducted a prospective, open-label, single-arm, multicenter trial to investigate the antitumor activity and safety of camrelizumab plus apatinib for patients with r/r PTCL.

## Methods

### Study design and patients

This was an open-label, multicenter, phase II, single-arm study. Patients were enrolled in six centers in China between October 2018 and March 2021. Patients with PTCL-NOS, AITL, ENKTL, and ALK-negative ALCL (ALK-ALCL), as defined by the WHO (2016), were eligible. The key inclusion criteria were: ≥18 years of age, relapsed or refractory to one or more systemic therapies, had measurable disease, had an Eastern Cooperative Oncology Group performance status of 0–2, had adequate bone marrow and organ function and had a life expectancy of at least 12 weeks. Refractory disease refers to the failure to achieve complete or partial remission after the latest treatment, whereas relapsed disease refers to confirmed disease progression after the latest treatment.

Patients were excluded if they had other prespecified NK/T-cell neoplasms or central nervous system infiltration. Additional exclusion criteria included a history of prior allogeneic HSCT; less than 90 days after autologous HSCT; previously used anti-PD-1, anti-PD-L1 or other ICIs; hemophagocytic syndrome at initial diagnosis (20); use of steroids (10 mg/day of prednisone or equivalent) or other immunosuppressants within 14 days before study treatment, or use of any conventional chemotherapy, radiation therapy and immunotherapy within 4 weeks before study treatment.

The study was approved by an independent ethics committee (2018YJZ34) and was conducted in accordance with the Declaration of Helsinki and Guidelines for Good Clinical Practice. All enrolled patients provided informed consent. This study was registered with ClinicalTrials.gov, NCT03701022.

### Treatment and assessments

All patients received camrelizumab 200 mg intravenously every 2 weeks and apatinib 500 mg orally once daily, 4 weeks as a cycle, until disease progression, unacceptable toxicity, or withdrawal of consent. Dose delay or reduction of apatinib (to 250 mg) was allowed. Dose delay for camrelizumab was allowed, and dose modification was not allowed. The maximum duration of dose delay was 8 weeks for camrelizumab and 4 weeks for apatinib. If the length of the dose delay exceeded the maximum duration, the treatment agent was discontinued. However, during initial treatment, more than two-thirds of patients experienced dose reduction or discontinuation due to apatinib-related adverse events (AEs). Therefore, the study protocol was amended to version 1.1 on May 18, 2020, and the starting dose of apatinib was set to 250 mg.

The response evaluation was performed according to Lugano 2014 response criteria. For the response evaluation, positron emission tomography-computed tomography (PET-CT) was recommended, while CT was allowed upon the patient’s request. Imaging assessment was performed every 8 weeks for the first 6 months, every 12 weeks from 6 months to 12 months, and every 16 weeks thereafter until disease progression or the end of study. Hyperprogression was defined as a tumor growth rate at the first assessment that is equal to or greater than twice the baseline. The safety records of this study included clinical symptoms, vital signs, physical examination, and laboratory tests. All AEs were monitored and documented until 90 days after the last dose. The grading of AEs was performed according to the Common Terminology Criteria for Adverse Events, version 4.03.

### Biomarker assessment

Tumor samples prior to receiving any therapy were used for biomarker analysis. PD-L1 immunohistochemistry was performed on formalin-fixed paraffin-embedded tumor biopsy samples using the PD-L1 IHC 22C3 pharmDx antibody (Dako, CA). Measurement of PD-L1 expression was based on the estimated percentage of PD-L1-stained cells.

### Endpoints

The primary endpoint was the ORR, defined as the percentage of patients with a confirmed complete response (CR) or partial response (PR) according to the Lugano 2014 response criteria. Secondary endpoints included duration of response (DoR, defined as the time from first documented objective response to disease progression or death from any cause); PFS, (defined as the time from the first dose to disease progression or death from any cause, whichever occurred first); overall survival (OS, defined as the time from the first dose to death from any cause); and the correlation between the expression of biomarkers such as PD1/PD-L1 and clinical outcomes.

### Statistical analysis

This phase 2 study aimed to discriminate a promising ORR of 40% from a ORR of 10% using a type I error of 0.05 and power of 80%. With this assumption, 13 evaluable patients were needed.

Patients who received at least one dose of either study drug were included for safety analysis and efficacy analysis. Descriptive statistics and the Clopper-Pearson method were used to summarize the proportion of patients with response and to calculate the 95% confidence intervals (CI), respectively. All time-to-event endpoints (PFS, DoR, and OS) were analyzed by the Kaplan–Meier method with a 95% CI. The 95% CI for the median times was estimated using the Brookmeyer-Crowley method. All statistical analyses were conducted using SAS 9.4 (SAS Institute Inc, Cary, NC, USA).

## Results

### Patients

**Figure 1:**
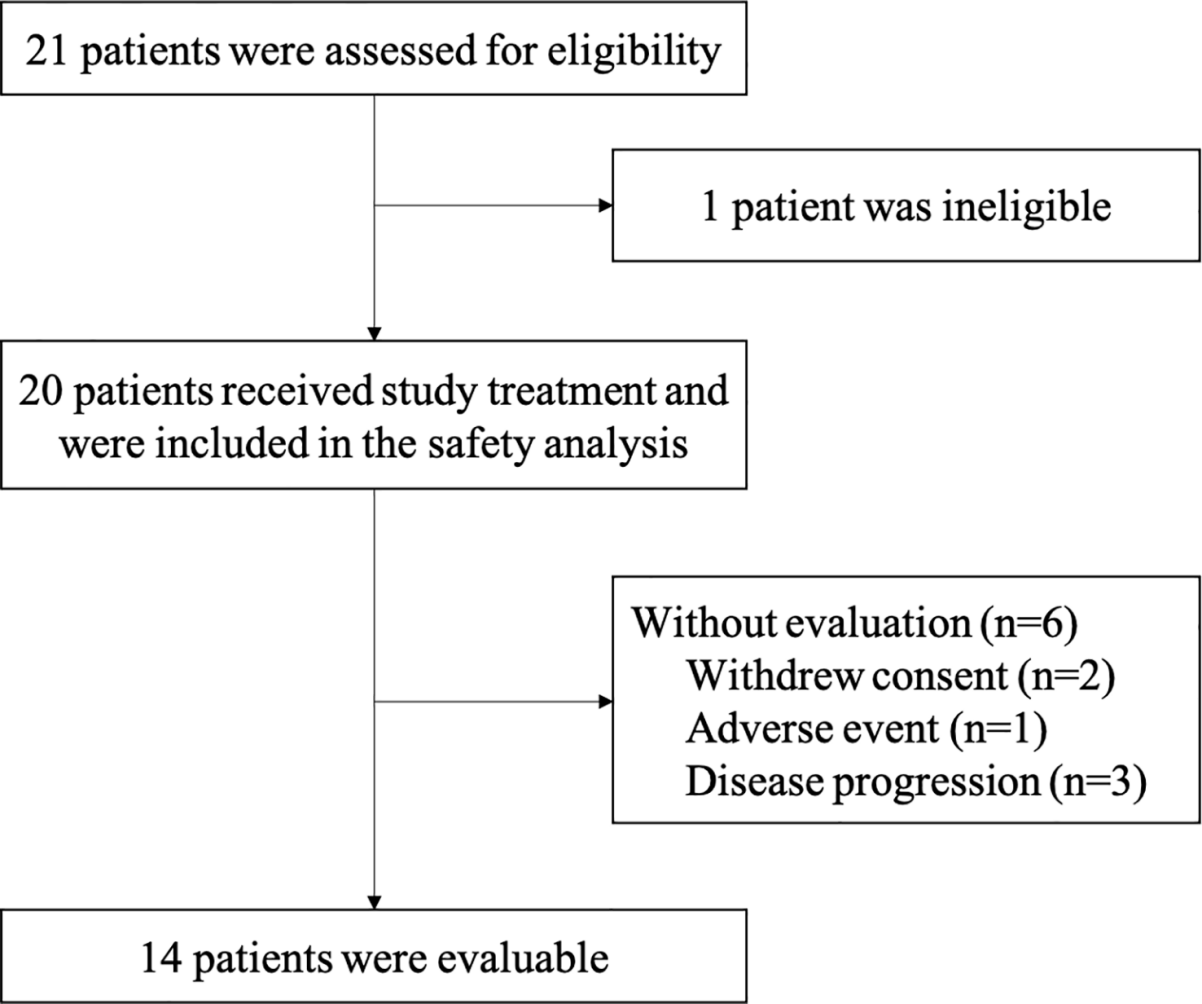
Patient flowchart.

Twenty patients were enrolled and received study medications in the study. A total of 15 patients received 500 mg of apatinib, and five patients received 250 mg. Six patients had early discontinuation without tumor response evaluation (one patient was due to toxicity after only one dose of camrelizumab; two patients withdrew consent, and three patients withdrew due to disease progression but not hyperprogression). At the cutoff date of March 4, 2022, the median follow-up was 27.2 months (range: 0.5 to 39.9 months), and three patients remained on treatment (**Figure 1**).

The median age of all patients was 52.5 years (range 22 to 66), and 13 patients (65%) were male. Seventeen (85%) had diagnosed stage III–IV disease. The most common subtypes were ENKTL (35%) and PTCL-NOS (30%). Patients were heavily pretreated before enrollment, with a median prior treatment line of 3 (range: 1-6). Most patients were assessed as being transplant ineligible because of chemotherapy resistance or rapid progression. Among the patients, 15 had refractory disease while five had relapsed disease. Two of seven patients with ENKTL had a PINK score of ≥2, while ten of 13 patients with other PTCL subtypes had an international prognostic index (IPI) score of ≥2. All seven patients with ENKTL had previously received asparaginase-based chemotherapy, and all patients with other subtypes of PTCL had received anthracycline-based chemotherapy. Eleven (55%) patients had previously received chidamide, and four (20%) patients had received autologous HSCT. The detailed baseline characteristics are shown in **Table 1**.

**Table 1:** Baseline characteristics of patients.

Characteristics	Patients (n=20)
Age, median (range)	52.5 (24-66)
Sex, n (%)	
Male	13 (65%)
Female	7 (35%)
Subtype	
ENKTL	7 (35%)
PTCL-NOS	6 (30%)
AITL	5 (25%)
ALK-ALCL	2 (10%)
PINK score for ENKTL (n=7)	
0	1 (14%)
1	4 (57%)
2	2 (29%)
IPI score for other types of PTCL (n=13)	
1	3 (23%)
2	5 (38%)
3	5 (38%)
Disease status, n (%)	
Relapsed	5 (25%)
Refractory	15 (75%)
Previous treatment lines, median (range)	3 (1-6)
Prior autologous HSCT, n (%)	4 (20%)
Prior Chidamide, n (%)	11 (55%)
Stage III/IV, n (%)	17 (85%)

ENKTL, extra nodal natural killer/T-cell lymphoma; PTCL-NOS, peripheral T-cell lymphoma, not otherwise specified; AITL, angioimmunoblastic T-cell lymphoma; ALK-ALCL, ALK negative anaplastic large cell lymphoma; IPI, international prognostic index; HSCT, hematopoietic stem cell transplant.

### Efficacy

**Figure 2:**
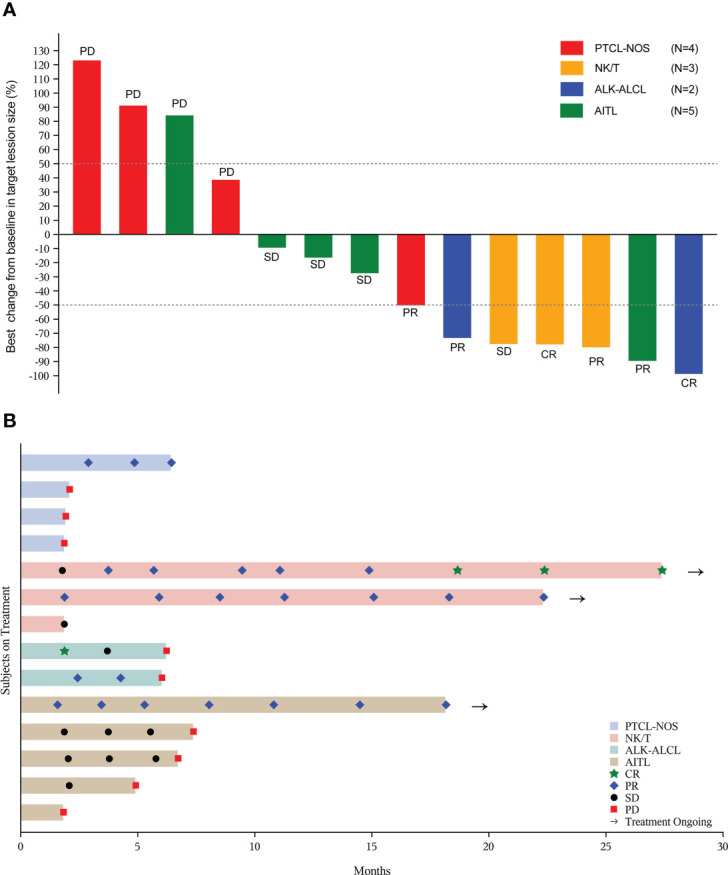
Tumor response in evaluable patients (n=14). **(A)** The best percentage changes from baseline in target lesions; **(B)** Treatment exposure and response duration. PTCL-NOS, peripheral T-cell lymphoma, not otherwise specified; NK/T, natural killer/T-cell lymphoma; ALK-ALCL, ALK negative anaplastic large cell lymphoma; AITL, angioimmunoblastic T-cell lymphoma; CR, complete response; PR, partial response; SD, stable disease; PD, progressive disease.

In total of 20 patients, 14 paitents were evaluable for efficacy, and the median treatment cycle of the combined regimen was 7 (range 2 to 21). For all patients, the ORR was 30% (6/20) (95% CI, 11.9% to 54.3%), with two patients (10%) achieving CR (one patient with ENKTL and the other with ALK-ALCL). The median DoR was not reached (NR) (range: 3.6 months to NR) (**Table 2**). Three patients achieved durable remission and remained on treatment (**Figure 2**).

**Table 2:** Tumor response of all patients.

Response	Patients (n=20)
Best of response, n (%)	
Complete response	2 (10.0%)
Partial response	4 (20.0%)
Stable disease	4 (20.0%)
Progressive disease	4 (20.0%)
Not evaluable	6 (30.0%)
Objective response rate, n (%) (95% CI)	6 (30.0%) (11.9%-54.3%)
Disease control rate, n (%)(95% CI)	10 (50.0%) (27.2%-72.8%)
Duration of Response, months, median (95% CI)	NR (3.6-NR)

NR, not reached; CI, confidence interval.

**Figure 3:**
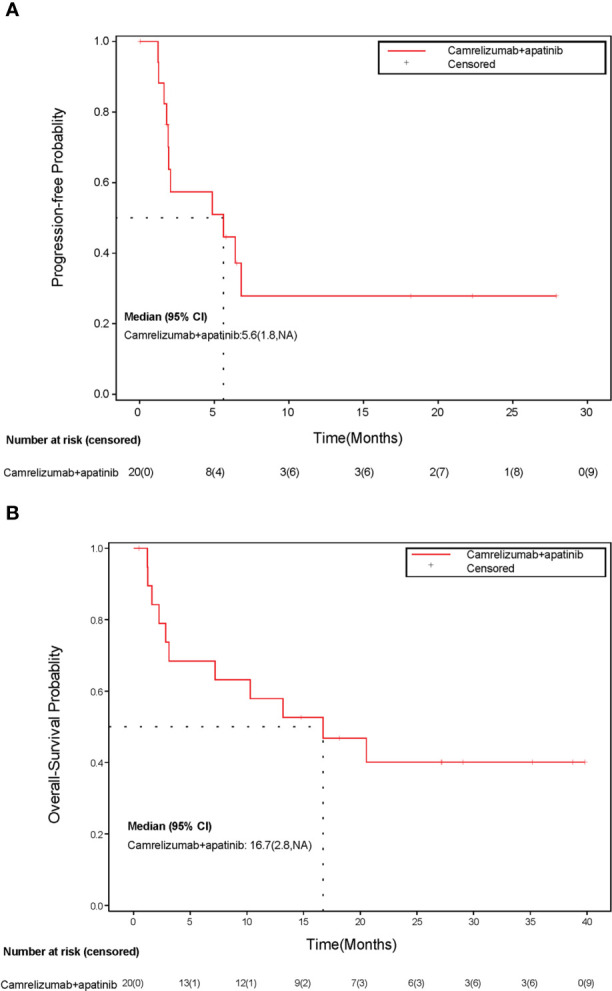
Kaplan-Meier curve of **(A)** progression-free survival and **(B)** overall survival. CI, confidence interval; NA, not available.

After a median follow-up of 27.2 months (range: 0.5 to 39.9 months), 11 (55%) of 20 patients died. The median PFS for all patients was 5.6 months (95% CI, 1.8 to NR) (**Figure 3A**), and the median OS was 16.7 months (95% CI, 2.8 to NR) (**Figure 3B**). The 1-year PFS and 1-year OS were 27.9% (95% CI, 8.1% to 52.2%) and 57.9% (95% CI, 33.2% to 76.3%), respectively.

### Biomarker analysis

**Figure 4:**
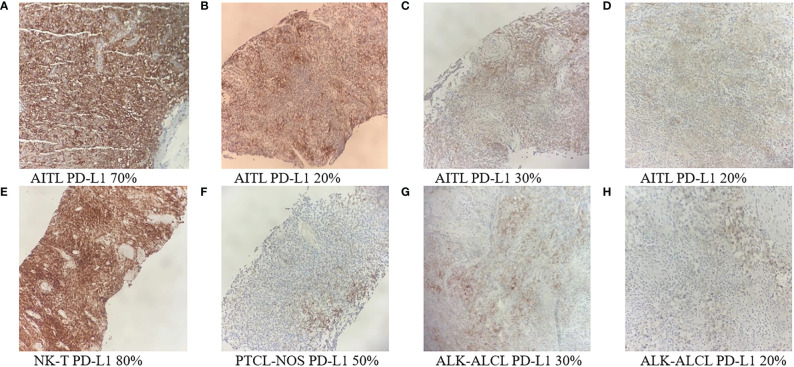
PD-L1 immunohistochemistry staining of tumor biopsy samples from eight PTCL patients **(A-H)** (Original magnification ×100). AITL, angioimmunoblastic T-cell lymphoma; ALK-ALCL, ALK negative anaplastic large cell lymphoma; ENKTL, extra nodal natural killer/T-cell lymphoma; PTCL-NOS, peripheral T-cell lymphoma, not otherwise specified.

Eight patients had available tumor biopsy samples for PD-L1 testing (**Figure 4**). All of them were positive for PD-L1 expression, and three (37.5%) had a PD-L1 ≥ 50% (**Table 3**). Patients with PD-L1 expression ≥ 50% had a numerically higher ORR (66.7% vs 40%). The two patients with the highest PD-L1 expression showed PFS of 18.2 and 22.3 months, respectively.

**Table 3:** Outcomes of eight patients with PD-L1 expression result.

Case number	Pathological subtype	Best overall response	PFS, months	PD-L1
1	AITL	Stable disease	6.5	20%
2	AITL	Stable disease	5.8	20%
3	AITL	Progressive disease	1.8	30%
4	AITL	Partial response	18.2	70%
5	ALK-ALCL	Partial response	5.7	20%
6	ALK-ALCL	Complete response	6.8	30%
7	ENKTL	Complete response	28.0	80%
8	PTCL-NOS	Progressive disease	2.0	50%

AITL, angioimmunoblastic T-cell lymphoma; ALK-ALCL, ALK negative anaplastic large cell lymphoma; ENKTL, extra nodal natural killer/T-cell lymphoma; PTCL-NOS, peripheral T-cell lymphoma, not otherwise specified; PFS, progression-free survival.

### Safety

A summary of toxicities possibly related to camrelizumab and/or apatinib that occurred in ≥ 10% of patients is presented in **Table 4**. The most common AEs of any grade were hyperlipidemia (30%), leukopenia (30%), fever (30%), hypokalemia (25%), neutropenia (25%), thrombocytopenia (25%) and increased lactate dehydrogenase (25%). The most commonly reported grade ≥ 3 AEs were hyperlipidemia (15%), hypokalemia (15%) and anemia (15%). No treatment-related deaths occurred.

**Table 4:** Treatment related adverse events occurred in ≥ 10% of patients.

Event, n (%)	Any Grade	Grade 1-2	Grade 3	Grade 4
Hyperlipidemia	6 (30%)	3 (15%)	2 (10%)	1 (5%)
Decreased white blood cell count	6 (30%)	5 (25%)	1 (5%)	0
Fever	6 (30%)	6 (30%)	0	0
Hypokalemia	5 (25%)	2 (10%)	2 (10%)	1 (5%)
Neutropenia	5 (25%)	3 (15%)	1 (5%)	1 (5%)
Thrombocytopenia	5 (25%)	3 (15%)	1 (5%)	1 (5%)
Increased lactate dehydrogenase	5 (25%)	5 (25%)	0	0
Increased bilirubin	5 (25%)	4 (20%)	1 (5%)	0
Anemia	4 (20%)	1 (5%)	3 (15%)	0
Hypertension	4 (20%)	2 (10%)	2 (10%)	0
Liver function injury	4 (20%)	3 (15%)	1 (5%)	0
Proteinuria	4 (20%)	3 (15%)	1 (5%)	0
Hypocalcemia	3 (15%)	2 (10%)	1 (5%)	0
Hyponatremia	3 (15%)	2 (10%)	0	1 (5%)
Hyperglycemia	2 (10%)	1 (5%)	1 (5%)	0
Increased γ-glutamyltransferase	2 (10%)	1 (5%)	1 (5%)	0
Increased alkaline phosphatase	2 (10%)	1 (5%)	1 (5%)	0
Hypohemoglobin	2 (10%)	2 (10%)	0	0
Increased alanine aminotransferase	2 (10%)	2 (10%)	0	0
Decreased albumin	2 (10%)	2 (10%)	0	0
Increased α-hydroxybutyrate dehydrogenase	2 (10%)	2 (10%)	0	0
Hyperuricemia	2 (10%)	2 (10%)	0	0

Two patients discontinued treatment due to AEs. One patient experienced grade 3 leukopenia and discontinued oral apatinib. Considering that PD-1 inhibitor monotherapy was not defined as a standard treatment, the patient withdrew from the study. Another patient discontinued the treatment due to grade 4 toxic epidermal necrolysis (TEN), which was relieved after steroid treatment.

## Discussion

To our knowledge, this is the first study to report the efficacy and safety profile of a PD-1 inhibitor combined with an antiangiogenic drug for r/r PTCL. In this study, the combination of camrelizumab and apatinib-treated r/r PTCL obtained an ORR of 30% and a CR rate of 10%. The median PFS and OS were 5.6 months and 16.7 months, respectively. The toxicity was tolerable, and no new safety signals were detected.

The prognosis of r/r PTCL is poor. Several studies have reported the outcomes of r/r PTCL at a population level, showing a median OS of less than 6 months (21, 22). Currently, optimal treatment for patients with r/r PTCL is not defined. For transplant-eligible individuals, salvage chemotherapy following autologous HSCT is appropriate. A phase 3 trial of CCTG LY.12 included 59 r/r PTCL patients who plan to receive autologous HSCT (23). All patients were randomly assigned to GDP (gemcitabine, dexamethasone, and cisplatin) or DHAP (dexamethasone, high-dose cytarabine, and cisplatin) prior to autologous HSCT and obtained a similar ORR (GDP: 38%; DHAP: 33%) after 2 cycles. One-year event-free survival (EFS) and OS were 16% and 28% for all patients, and two-year EFS and OS were 21% and 42% for patients who received autologous HSCT (n=19). Several previous studies have shown that approved monotherapies, such as chidamide, romidepsin or pralatrexate, have an ORR of 25%-29% in r/r PTCL patients (6, 8, 9). PD-1 inhibitors have been confirmed to have remarkable efficacy in solid tumors and Hodgkin lymphoma. However, a small pilot study reported modest clinical activity in r/r PTCL patients. Twelve r/r PTCL patients treated with nivolumab showed an ORR of 33% (11). The median PFS and OS were 1.9 months and 7.9 months, respectively. More notably, 4 patients experienced hyperprogressive disease. Another study showed an ORR of 33% (5/15) for pembrolizumab monotherapy in r/r mature T-cell lymphoma, which was terminated after interim futility analysis (24). In China, a study with a larger sample size reported that 89 r/r PTCL patients treated with geptanolimab, an anti-PD-1 antibody, had ORR and CR rates of 40.4% and 14.6%, respectively (12). While hyperprogressive disease was not observed in our study, the efficacy of combination therapy does not seem to be markedly superior to that of PD-1 inhibitor monotherapy.

It is generally believed that the pathogenesis of ENKTL is closely related to Epstein−Barr virus (EBV) infection. EBV can drive LMP1 to upregulate PD-L1 expression on T lymphocytes (25). Therefore, blockade of the PD-1/PD-L1 axis is a potential mechanism for the treatment of ENKTL. In a retrospective analysis from Asia, 7 patients with r/r ENKTL were treated with pembrolizumab and achieved an ORR of 100% (26). Recently, a phase 2 study of PD-1 inhibitor sintilimab in 28 patients with r/r ENKTL showed an ORR of 75% and a disease control rate (DCR) of 85.7% (27). Additionally, a phase 2 study evaluating avelumab, a PD-L1 inhibitor, in 21 patients with r/r ENKTL showed a CR rate of 24% and an ORR of 38% (28). In our study, three of seven r/r ENKTL patients could be evaluated for efficacy; all three had disease control and two achieved duration response. Although the potential benefits of combination therapy could not be fully assessed due to the small sample size of evaluable patients with ENKTL, investigating PD1 inhibitor combination therapy remains a crucial avenue for future research.The prognosis of PTCL-NOS and AITL is almost the worst among the subtypes. In this study, the efficacy was modest in these two types, with ORRs of 16.7% and 20%, respectively. However, it is noteworthy that one AITL patient who was in a poor condition before enrollment improved significantly after treatment, and the effect was persistent. Perhaps it is meaningful to further investigate the tumor immune microenvironment of this case. ALK-ALCL is a relatively rare subtype of PTCL, and the prognosis is not optimistic. At present, there are no targeted therapeutic agents for ALK-ALCL. In our study, both patients obtained an objective response but had dismal long-term remission. Larger studies are needed to confirm the findings.

During tumor growth, abnormal vasculature formation occurs, which creates a hypoxic microenvironment that promotes the differentiation of immunosuppressive cells from inflammatory cells, hindering the infiltration of T cells and other immune cells into the tumor microenvironment (29). VEGF, a major angiogenic cytokine induced by hypoxia, plays a significant role in immunosuppression, which implies that antiangiogenic therapy may be a potential way to enhance immunotherapy. Preclinical studies have shown that the combination of antiangiogenic agents with immunotherapy has synergistic antitumor effects (15). Antiangiogenic agents can reprogram the TME by normalizing tumor vasculature, increasing T cell recruitment and infiltration, and promoting antigen presentation. A retrospective study that evaluated seven patients with Hodgkin lymphoma who failed immunotherapy found that the combination of camrelizumab and apatinib achieved an ORR of 86% (30). This suggests that antiangiogenic agents may have the ability to improve immunosuppression. Currently, numerous clinical studies have demonstrated the promising antitumor effects of combining ICIs with antiangiogenic agents in various solid tumors, such as lung cancer, hepatocellular carcinoma, colon adenocarcinoma, gastric cancer, melanoma, renal cell carcinoma, and breast cancer (31–33).

Preclinical studies have shown that low-dose apatinib may upregulate PD-1 expression on tumor-infiltrating immune cells more effectively than full-dose apatinib, increase immune effector cell infiltration into tumors, and enhance the antitumor activity of PD-1 inhibitors (34, 35). Based on these findings, the initial dose of apatinib in this study was 500 mg/d, but most patients experienced poor tolerance and a high incidence of treatment-related AEs. The amended study protocol reduced the dose of apatinib to 250 mg. Nevertheless, our study did not find a significant synergistic effect of combination therapy, which may be attributed to the complex immune microenvironment of PTCL, and antiangiogenic agents may not be sufficient to reverse the immunosuppressive state of the TME. Besides, this may also be limited by the small sample size of evaluable patients in our study. A retrospective study suggested that the combination of PD-1 blockade, antiangiogenic agent, pegaspargase and radiotherapy was feasible for localized NK/T cell lymphoma (36). In addition, a single-arm phase II trial with the combination of camrelizumab plus low-dose apatinib and pegaspargase followed by radiotherapy for newly diagnosed stage I/II NK/T cell lymphoma is ongoing (NCT04366128). These studies may provide more evidence of the combination of PD-1 blockade and antiangiogenic agent for patients with PTCL.

The combination of low-dose apatinib and camrelizumab did not result in unexpected toxicity. Except for 2 patients who discontinued treatment due to AEs, the remaining patients tolerated treatment well. Hyperlipidemia, hypertension, proteinuria, and hematologic toxicity observed in this trial were considered apatinib-related, while hyperthyroidism was considered camrelizumab-related. Other AEs were probably due to the combination therapy. The incidence of AEs of the combination therapy was no higher than that of monotherapy (14, 16). Reactive cutaneous capillary endothelial proliferation (RCCEP), which is common with camrelizumab monotherapy, occurred in 67% to 97% of patients in previous studies. Due to the antiangiogenic effect of apatinib, RCCEP was not observed in patients treated with this combination therapy, and only one patient developed RCCEP after discontinuation of apatinib. According to previous studies, hand-foot syndrome is a common AE related to apatinib, with an incidence of 20%-53% (37). In this study, probably due to the low dose of apatinib, only one patient who received 500 mg developed grade 1 hand-foot syndrome. TEN is a rare and life-threatening cutaneous complication of immunotherapy. One patient in this study developed TEN but eventually recovered, and we had no evidence that the combination therapy could increase the incidence of TEN. Further exploration may be needed.

There are several limitations. First, this study consisted of a relatively small sample size. Second, it was a single arm without a control group. Further study is needed to confirm our findings.

Camrelizumab plus apatinib had preliminary clinical activity and manageable toxicity in patients with r/r PTCL. Further investigations are necessary to examine the potential of this combination regimen in treating patients with PTCL.

## Data availability statement

The raw data supporting the conclusions of this article will be made available by the authors, without undue reservation.

## Ethics statement

The studies involving human participants were reviewed and approved by Ethics Committee of Peking University Cancer Hospital & Institute. The patients/participants provided their written informed consent to participate in this study.

## Author contributions

YL, YX, YS, SZ, XZ, HL, JWW and JBW conceptualized and designed the study. YL, YX, YS, SZ, XZ, HL, JWW, JBW, YT, WZ, ZY, LP, CZ, MW and JZ acquired the data. All authors contributed to the article and approved the submitted version.
